# A possibility to infer frustrations of supported catalytic clusters from macro-scale observations

**DOI:** 10.1038/s41598-024-54485-z

**Published:** 2024-02-15

**Authors:** Alexander Korobov

**Affiliations:** Materials Chemistry Department, V. N. Karazin Kharkiv National University, Kharkiv, 61022 Ukraine

**Keywords:** Heterogeneous catalysis, Nanoparticles, Computational science

## Abstract

Recent experimental and theoretical studies suggest that dynamic active centres of supported heterogeneous catalysts may, under certain conditions, be frustrated. Such out-of-equilibrium materials are expected to possess unique catalytic properties and also higher level of functionality. The latter is associated with the navigation through the free energy landscapes with energetically close local minima. The lack of common approaches to the study of out-of-equilibrium materials motivates the search for specific ones. This paper suggests a way to infer some valuable information from the interplay between the intensity of reagent supply and regularities of product formation.

## Introduction

The concept of active centre (site) of heterogeneous catalysts began to take shape in publications of 1920th–1930th, in particular in^1–3^. In their retrospective reading it catches the eye that practically the whole spectrum of possibilities is covered. For example, in considering nickel as the catalyst it was suggested that active Ni atom initially “held to the rest of the solid surface by a single constraint, but can be detached therefrom … and become for the time being a gaseous (nickel) atom”^3^. In modern terms this is a single-atom dynamic active centre (as it was seen in 1925; compare with current results and ideas^4^).

Nonetheless, until relatively recently a clear predominance belonged to the concept of stable active centre nearly invariable under operando conditions. To a certain extent this reflected the available research methods. And though an impressive progress is reached in examining the atomic structure of catalytic centres, in kinetic models they usually appear simply as * or z; i.e. the atomic-level knowledge is poorly translated to the next level.

Recent development of heterogeneous catalysis, both experimental and theoretical, significantly modified the understanding of the reaction centre^5–9^. The active centre under operando conditions may significantly differ from its “precursor” as part of just-prepared catalyst. Even more, the centre may appear under operando conditions and may be dynamic (labile, fluxional) during the catalytic process^10–15^. If cluster catalysis is concerned^16–20^, an important feature is that the most active isomer not obligatory corresponds to the lowest minimum at the potential energy surface (PES).

In this context, an understanding start to emerge that, under certain conditions, dynamic centres can in addition be frustrated. The term “frustration” here implies a large set of low-energy isomers accessible at operando conditions and degenerated local minima of FES separated with surmountable barriers. Under these conditions there is a chance that one or another isomer will be dynamic during the elementary catalytic reaction. The characteristic times of interconversion and reaction will be comparable. The realization of this chance strongly depends on particular operando conditions and is difficult to register.

Here heterogeneous catalysis borders on a rapidly developing and promising field of out-of-equilibrium materials^21–26^. Possible frustrations are of interest for two main reasons. First, peculiar catalytic properties and tunability are expected. Second, most important, expectation is the higher functionality of catalytic systems. It is associated with the navigation through the ensembles of energetically close local minima of FES. The other side of the coin is the lack of general approaches in exploring out-of-equilibrium materials. For the present the available knowledge is restricted to conjectures and scattered results of real and computational experiments^27–34^. Thus, structural dynamics was experimentally confirmed for ultrasmall Au clusters supported on CeO_2_ in the reaction of CO oxidation^30^. Frustrated structures revealed for tricopper metallohelicate^32^ and layered hydrogen boride^33,34^. Their catalytic potential is under study. Purely computational results demonstrate the structural dynamics of Pt_13_ clusters in hydrogen evolution reaction and methane activation^29^, and also for Pt_7_ clusters supported on Al_2_O_3_^28^.

An important aspect, in particular, is the coupling of the dynamic structural evolution of active sites with elementary reactions. Within the bottom-up approach the main stumbling block in exploring frustrated catalysts is, seemingly, the transition from multi-dimensional PES to reasonably-dimensional FES since the errors for such involved systems is comparable with studied properties. One more problem is to take into account the internal degrees of freedom of the frustrated catalytic centre and their coupling with elementary reactions. (Since the catalytic centre and elementary reactions are no longer separate entities). Within the top-down approach generally there are quite sophisticated experimental methods both at the atomic level and kinetic level. But results obtained at these levels are disjoint in the sense that in kinetic models the catalytic centre as a rule static (* or z). Accordingly, if it is actually dynamic, it is problematic to infer this from kinetic data. Also, the conclusions within this approach rely on the scope of published results.

With this in mind, any (particular) steps towards an insight into the nature and functionality of frustrated catalytic systems seem to be in demand. Previously, simple lattice models were used to demonstrate that the dynamic behaviour of the active centre does not reduce the stability of catalytic system^35,36^ and that in the case of frustrated catalytic system its efficiency depends on the coverage in a characteristic way^37^. This paper shows that for frustrated catalytic system there should be a certain characteristic dependence of the regularity of product formation on the intensity of reactant supply, different from simpler cases.

The most straightforward example is the parallel monomolecular reactions in which reactant molecules may generally turn into two different products. Typically these are isomerization or elimination reactions, as illustrated below.
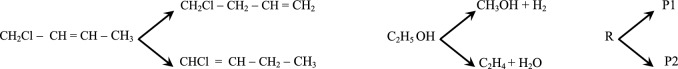


Note that only the reagent concentration enters the kinetic mass action law. Accordingly, the formalization of these reactions will be the same regardless of whether one molecule of the product is formed or two. If the reactant molecule R reaches the conventional static catalytic centre on the substrate, there are three possibilities. (i) With some probability *p*_1_ it turns into first product P1 with subsequent desorption and release of the active centre. (ii) With some probability *p*_2_ it turns into second product P2 with subsequent desorption. (iii) With some probability (1–*p*_1_–*p*_2_) it returns to the reactant phase (to the substrate or into gas phase). The result is the mixture of two products. Their ratio and the efficiency of the active centre are determined by *p*_1_ and *p*_2_, and are not sensitive to the supply intensity.

**Figure 1:**
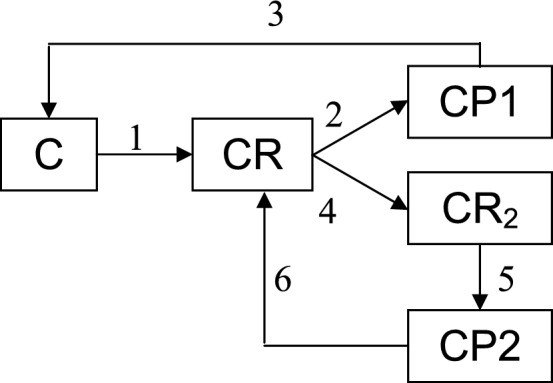
Graph representation of the feasible behaviour of frustrated catalytic centre in the case of two parallel monomolecular reactions; *C* cluster, *R* reactant, *P*1 and *P*2 products, numbers above arrows enumerate elementary stages.

If the reactant molecule R reaches a dynamic frustrated catalytic centre (e.g. small metal cluster), different scenarios are generally possible. Within the previously suggested simple lattice model^37^ it is as follows. The adsorption of the reactant molecule R changes the properties of the cluster C; actually an intermediate CR is formed. Its energy landscape is assumed to be frustrated which set the stage for highly selective formation of product P1 due to proper coupling of the elementary reaction with the internal degrees of freedom of the cluster. Note that at this stage there is no favourable way to second product P2. The probability and time of this transformation is determined by the corresponding minima on the free energy landscape. During this period of time the intermediate CR have a chance to adsorb one more reactant molecule and form another intermediate CR_2_. Its energy landscape is also frustrated and is highly favourable for the formation of the second product P2. The way to P1 is much less favourable. Note that after the desorption of P2 the intermediate CR is formed. Its further fate depends on the intensity of the reactant supply. Under intensive enough supply one more reactant molecule manages to join it with the formation of CR_2_. Under moderate supply P1 is formed and the cluster is released. The second reactant molecule in CR_2_ may be treated in this context as a chemical fuel which keeps the catalytic centre in a more active state. This may be represented in terms of graphs (Fig. 1).

Within the above model the result of the catalytic process depends on the intensity of reactant supply and may be of three types. In the diffusion regime (supply of low intensity) mainly P1 is formed. In the kinetic regime (supply of high intensity) mainly P2 is formed. In the intermediate regime both products are formed in the ratio symbatic with the supply. In the first case the catalytic centre spend some time in the weighting mode. In the second case the reactant molecules are always available.

When small active centres are concerned, some nuances of their supply with reactants should not be overlooked. Reactant molecules may arrive directly from the gas phase or via the substrate. Generally, both possibilities can occur simultaneously. But in practice one way is more efficient than the other. If the active centre is hospitable to reactant molecule (suitable sticking coefficient and dynamic steering), then as dense as possible situation of centres on the substrate is favourable. The substrate itself in this case is just the carrier of active centres. But usually its roles are more varied. In many cases the substrate is more suitable for adsorption. Also, it may pre-activate the reactant molecule since its adsorbed state differs from the state in the gas phase. In addition, the substrate provides the possibility to accumulate some reactant molecules in the nearest vicinity of the active centre, which is important in the present context. The direct hit of the reactant molecule into the active centre and its activated jump towards the centre from the neighbouring substrate site considerably differ in energy and other characteristics, as well as in the scatter of these characteristics. These roles may be useful for tuning catalytic systems. If all or some of them are relevant, the reasonable capture zone is required, which determines the desirable density of the active centres on the substrate. Note, however, that it is not always experimentally possible to provide the preassigned density and order of active centres.

One more peculiarity of small active centres is the need to take the product desorption into account in kinetic models. (In simpler cases desorption is considered to be instantaneous and is not included into models). The time interval between the formation of product molecule and its escape from the active centre may appear to be significant portion of the total internal time of the dynamic centre. Also, if the product molecule escapes to the substrate, it is no longer influence the active centre but may shield it from subsequent reactant molecules.

**Figure 2:**
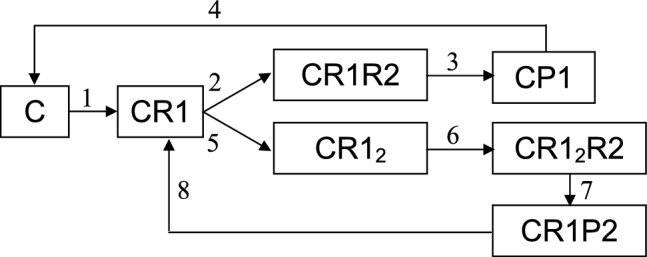
Graph representation of the feasible behaviour of frustrated catalytic centre in the case of two parallel bimolecular reactions; *C* cluster, *R*1 and *R*2 reactants, *P*1 and *P*2 products, numbers above arrows enumerate elementary stages.

In passing to bimolecular case, represented mainly by addition reactions,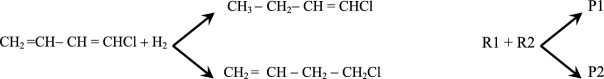 the picture becomes much more diverse. Note that whereas true monomolecular and bimolecular reactions are not very common, monomolecular and bimolecular elementary stages dominate in the networks of involved reactions. This is what determines main interest in them. When supported cluster catalysis is concerned, additional nuances appear. Two reagents may join the cluster in different order, the number of intermediates increases, the cycles on the graph become longer. One possibility is illustrated in Fig. 2. In comparison with Fig. 1 the number of intermediates is doubled. Each of them is assumed to have unique frustrated structure favourable for the formation of corresponding product due to the proper coupling of reaction with internal degrees of freedom of the cluster. Both cycles become one vertex longer, but the topology of graphs is similar.

In spite of all nuances, the dependence of the product formation on the supply intensity is basically preserved: product P1 dominates in the diffusion regime, product P2 dominates in the kinetic regime, and in the intermediate regime the ratio of products is symbatic with the intensity of reactant supply.

This similarity of two schemes in the interplay between the supply intensity and product formation is not occasional. Note that there is no direct collisions between R1 and R2 (as in conventional bimolecular reactions); reagents enter the play one by one. Basically, this similarity holds for other, more complex, kinetic schemes.

How do these general considerations on potential opportunities relate to chemical reality? It is very easy to provide examples of parallel mono- and bimolecular reactions. It is quite possible to find examples of clusters with frustrated free energy landscapes. But it is really a challenge to pick up the key to the lock. Even when the aim is to find model reactions for probing one or another catalyst for frustration. Then, what are main reasons to take this challenge. The brief answer is the hope to identify dynamic frustrated catalysts and to benefit from the control of their functionality. Note some similarity of discussed catalysts with enzymes, namely in respect of conformational dynamics. Enzymes are known to be highly efficient but not adaptive and not operating at high temperatures. Frustrated catalysts are seen as promising in bridging high functionality with high tunability and operating temperature. This strongly motivates to look for even smallest insight into these out-of-equilibrium systems, for which currently there are no general approaches and guidelines.

In the present paper the most simple model is considered, i.e. a metal cluster on a well-prepared surface (e.g. single crystal face). In reality a dynamic frustrated catalytic system may possess more involved active centre (composed of various atoms) as well as more involved substrate (defective, doped, porous, etc.). Small (1–2 nm) Ni clusters on frustrated hydrogen boride sheets is a thought-provoking example^33,34^. Accordingly, there is no unambiguous detailing of the term symbatic in discussing the interplay between supply intensity and regularities of product formation. It is next to impossible to provide ideal supply. (Note in this regard works aimed at targeted control of the microenvironment of the catalytic centre^38,39^.) What is possible is to provide reproducible supply and to register the performance of the catalytic system. Regularities of this kind are relatively seldom examined and reported. Hopefully, this publication will add arguments in favour of potential value of such information and will motivate the replenishment of relevant databases.

**Figure 3:**
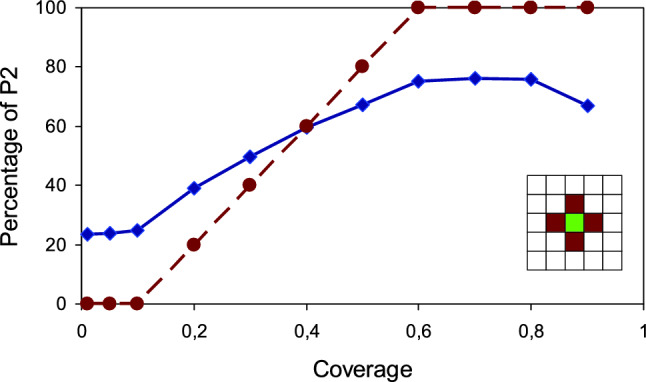
Supply-performance curve for bimolecular reactions (Fig. 2); kMC results are in blue, corresponding ideal curve is in brown. The insert shows the central part of the lattice, the catalytic centre (green) and its border (brown).

As an example, Fig. 3 shows kMC results for the lattice model corresponding to bimolecular reactions. Its main distinctive feature is that the central site of the *L* × *L* square lattice represents the dynamic frustrated catalytic centre. The involved behaviour of this site is described by the graph shown in Fig. 2. In particular it is characterised by internal time (counted in beats) and two pairs of energetically close minima separated by barriers surmountable under operando conditions. This special site has four-site border from which it takes reagent molecules R1 and R2. The rest of sites simulate the capture zone of the catalytic centre. The Langmuir–Hinshelwood supply mode is assumed: both reagents adsorb from the gas phase and then diffuse towards the catalytic centre. The ratio of reagents is stoichiometric. Sticking coefficients and diffusion coefficients are assumed to be equal for R1 and R2.

Details of the kMC simulation are as follows. The lattice size is taken to be 11 × 11 with periodic boundary conditions. Time is measured in Monte Carlo steps (MCS). One MCS is time during which each site of the lattice is visited once on the average; one MCS equals *L* × *L*. The simulation starts from empty lattice. At the first stage reagents R1 and R2 are randomly adsorbed in equal quantities at the lattice sites (except the catalytic centre) to provide the set value of the coverage θ. In what follows this value is kept constant. Once the required value of θ is reached, the catalytic centre start to absorb reagents from its four-site border in accordance with corresponding graph (Fig. 2). Here one beat is taken to be equal to one MCS. Then the duration of the longest rout on the graph r1 (1–2–3–4) equals 15 MCS, duration of *r*2 (1–5–6–7–8) equals 12 MCS, duration of *r*3 (2–3–4) equals 8 MCS, and the shortest rout *r*4 (5–6–7–8) equals 5 MCS. As in^37^, results for each value of coverage θ are the average of 10^6^ runs and *ad hock* parameters are the same.

Three sections are clearly distinguished on the curve: they correspond to kinetic, intermediate and diffusion modes. Dotted line sketches the unattainable ideal case. Note that with the given parameters the maximum selectivity is 80% and the kinetic mode cannot be kept at coverages exceeding 0.8. Both these features are because of the peculiarities of the supply; first of all because of the considerable unavoidable fluctuations of the border coverage. The diffusion mode is reached at very low coverages. Usually this range of substrate coverage is of no interest, but in the present context it bears sufficient information. The shape of the curve may be considerably closer to the ideal by increasing the border from four to eight sites. But when small catalytic centres (e.g. small metal clusters) are concerned, eight-site border seem somewhat unrealistic, and also the important role of fluctuations is not so obvious.

To conclude, the main distinctive feature of the suggested model is the catalytic centre represented in terms of graph which models its internal degrees of freedom. Within this model, a tree-section characteristic shape of the supply–performance curve is indicative of the frustration of the catalytic centre. Sections correspond to diffusion, mixed, and kinetic modes. However, three caveats need to be made. The diffusion mode is reached at very low coverages, uninterested in applied respect. The same adsorption and diffusion parameters are assumed in the whole range of coverages. The use of boundary conditions implicitly implies the regular situation of catalytic centres on the support. Since the targeted control of the microenvironment of catalytic centres is just start to develop, these preconditions are difficult to control. With this in mind, the results presented may sooner come in handy in the framework of catalytic informatics^40–43^. One possibility is the ML-assisted literature data mining^42^. This can be supplemented by generating computational training data sets with the use of suggested lattice model. Hopefully, these and other relevant steps will make a worthy contribution to exploring very promising but very challenging field of frustrated catalytic systems.

## Data Availability

No datasets were generated or analysed during the current study.
